# Antisense-Based Progerin Downregulation in HGPS-Like Patients’ Cells

**DOI:** 10.3390/cells5030031

**Published:** 2016-07-11

**Authors:** Karim Harhouri, Claire Navarro, Camille Baquerre, Nathalie Da Silva, Catherine Bartoli, Frank Casey, Guedenon Koffi Mawuse, Yassamine Doubaj, Nicolas Lévy, Annachiara De Sandre-Giovannoli

**Affiliations:** 1 Aix Marseille Université, INSERM, GMGF UMR_S 910, 13385 Marseille, France; Karim.HARHOURI@univ-amu.fr (K.H.); Claire.NAVARRO@univ-amu.fr (C.N.); camille.baquerre1@orange.fr (C.B.); Nathalie.DASILVA@univ-amu.fr (N.D.S.); catherine.bartoli@univ-amu.fr (C.B.); nicolas.levy@univ-amu.fr (N.L.); 2 Royal Belfast, Pediatric Cardiology, Hospital for Sick Children, Belfast BT9 7AB Northern Ireland; frank.casey@belfasttrust.hscni.net; 3 CHU Sylvanus Olympio de Lomé, Unité de Génétique Humaine, Lomé BP 1515, Togo; julesblack@yahoo.fr; 4 Département de Génétique Médicale, Institut National d’Hygiène, 11400 Rabat, Morocco; y.doubaj@gmail.com; 5 Departement of Medical Genetics, la Timone Children’s Hospital, APHM, 13385 Marseille, France

**Keywords:** HGPS-like, MAD-B, antisense oligonucleotides, Progerin, Prelamin A Δ90, Prelamin A Δ35

## Abstract

Progeroid laminopathies, including Hutchinson-Gilford Progeria Syndrome (HGPS, OMIM #176670), are premature and accelerated aging diseases caused by defects in nuclear A-type Lamins. Most HGPS patients carry a de novo point mutation within exon 11 of the *LMNA* gene encoding A-type Lamins. This mutation activates a cryptic splice site leading to the deletion of 50 amino acids at its carboxy-terminal domain, resulting in a truncated and permanently farnesylated Prelamin A called Prelamin A Δ50 or Progerin. Some patients carry other *LMNA* mutations affecting exon 11 splicing and are named “HGPS-like” patients. They also produce Progerin and/or other truncated Prelamin A isoforms (Δ35 and Δ90) at the transcriptional and/or protein level. The results we present show that morpholino antisense oligonucleotides (AON) prevent pathogenic *LMNA* splicing, markedly reducing the accumulation of Progerin and/or other truncated Prelamin A isoforms (Prelamin A Δ35, Prelamin A Δ90) in HGPS-like patients’ cells. Finally, a patient affected with Mandibuloacral Dysplasia type B (MAD-B, carrying a homozygous mutation in ZMPSTE24, encoding an enzyme involved in Prelamin A maturation, leading to accumulation of wild type farnesylated Prelamin A), was also included in this study. These results provide preclinical proof of principle for the use of a personalized antisense approach in HGPS-like and MAD-B patients, who may therefore be eligible for inclusion in a therapeutic trial based on this approach, together with classical HGPS patients.

## 1. Introduction

Progeroid laminopathies are a recently discovered group of accelerated aging syndromes caused by altered A-type Lamins processing [1]. Lamins A and C (hereafter called Lamin A/C) are encoded by the *LMNA* gene through physiological alternative splicing; Lamin A being encoded by exons 1–12 and Lamin C by exons 1–10. The use of a splice site within exon 10 leads to the production of Prelamin A-encoding transcripts [2,3]. Together with B-type Lamins, Lamin A/C are major components of the nuclear Lamina, a protein meshwork located underneath the inner membrane of the nuclear envelope and dispersed through nuclear matrix [4]. Lamin A is synthesized as a precursor protein, called Prelamin A, that undergoes a complex maturation process. Prelamin A contains a conserved C-terminal CaaX motif (C, cysteine; A, aliphatic; X, any amino acid) that undergoes farnesylation upon the action of a farnesyl-transferase [5]. Following farnesylation, the three C-terminal amino acids (aaX) of Prelamin A are cleaved and the new C terminus is methylated. This cleavage event does not require the endopeptidase ZMPSTE24 in physiological conditions [6]. Subsequently, farnesylated Prelamin A undergoes a second endoproteolytic cleavage, which, on the contrary, is uniquely mediated by ZMPSTE24 in humans, removing the last 15 C-terminal residues, including the farnesylated Cysteine residue. This multistep processing physiologically yields mature Lamin A that contains no farnesyl moiety [7]. Mutations in the *LMNA* gene have been described to cause HGPS (Hutchinson-Gilford Progeria Syndrome) [8,9], an extremely rare genetic disorder that affects 1 in 4–8 million newborns worldwide. HGPS patients generally appear normal at birth, but prematurely develop growth retardation and clinical signs recalling physiological aging including thin skin with hyperpigmented lesions, loss of subcutaneous fat, alopecia, osteoporosis and severe and generalized arteriosclerosis leading in most cases to myocardial infarction and death at the mean age of 13.5 years [10]. Most HGPS patients carry a de novo point mutation in exon 11 (c.1824C > T, p.G608G). This mutation activates a cryptic donor splice site within exon 11 that leads to the in-frame deletion of 150 nucleotides at its C-terminal domain comprising the ZMPSTE24 cleavage site. Accordingly, incomplete processing of Prelamin A results in the expression of a truncated and toxic Lamin A precursor called Prelamin A Δ50 or Progerin. As a result, Progerin remains permanently farnesylated, and it accumulates in patients’ cells nuclei exerting toxic effects [8,9,11]. Other de novo heterozygous mutations of the *LMNA* gene have been identified in patients with variable features of HGPS. These mutations affect exon 11 splicing leading to the production of Progerin and/or other truncated Prelamin A isoforms (Δ35 and Δ90) at the transcriptional and/or protein level [12,13,14,15,16]. In particular, Prelamin A Δ90 transcript excludes the 270 nucleotides of exon 11 because of the abolition of the normal donor splice site. The resulting deletion is predicted to preserve the Prelamin A open reading frame (r.[=, 1699_1968del], p.(Gly567_Gln656del)). The mutation responsible for the production of Prelamin A Δ35 generates a conservative substitution of serine with threonine and activates a cryptic splice site resulting in expression of Lamin A with a 35 amino acid truncation (r.[=, 1864_1968del], p.[Thr623Ser, Val622_Gln656del]); patients are affected with “Progeria-like” syndromes and have thus been recently named “HGPS-like” patients [12]. Patients affected with mandibuloacral dysplasia type B (MAD-B), carry biallelic mutations in the *FACE1/ZMPSTE24* gene. As a consequence, the last cleavage step of Prelamin A maturation is only partially performed, leading to the accumulation of wild type, permanently farnesylated and toxic Prelamin A isoforms [17,18,19].

To date, several therapeutic strategies have been proposed to either: (a) counteract the toxic effects of Prelamin A isoforms accumulated in patients’ cells [20]; or (b) reduce the quantities of aberrant Prelamin A isoforms [21,22]. (a) The first attempts aiming to decrease Prelamin A toxicity by reducing its farnesylation, used Farnesyl-transferase inhibitors (FTIs) [23,24] or, more globally, its prenylation, used zoledronate and pravastatin [6]. (b) The second approach aimed to reduce Prelamin A quantities by blocking the cryptic splice site leading to Progerin mRNA production using morpholino antisense oligonucleotides (AON) [1,25,26]. Morpholinos are small modified oligonucleotides that modulate splicing via steric blockade of pre-mRNA targets [27]. (c) Subsequent attempts aimed to reduce downstream toxic effects of Prelamin A accumulation, by acting on inflammation or IGF-1 levels [28,29]. Other therapeutic strategies for Progeria could be considered, such as gene therapy approach (CRISPR), cell-based replacement therapies, particularly targeting the vascular tissue, using either matching wild-type cells or tissue-stem cells generated from genetically corrected HGPS iPS cells, RNA Therapy based on RNA interference, or the antioxidant based approach [30,31].

The efficiency of AON in blocking the aberrant *LMNA* splicing leading to Progerin production has been previously proven in vitro on classical HGPS patient’s cells and in vivo on a knock-in mouse model (*Lmna^G609G/G609G^*) closely mimicking Hutchinson-Gilford Progeria [25]. In the latter mouse line, Osorio et al. tested the combined administration of two AONs: “MmEx11” targeting the exon 11 aberrant splice site activated by the Progeria mutation in order to hamper its use, and “MmEx10”, targeting the physiological exon 10 splice site, in order to reinforce the action of the first AON, by shifting splicing events towards Lamin C mRNAs while reducing the production of all Lamin A isoforms, including Progerin.

To increase the uptake of oligonucleotides by tissues, Vivo-morpholinos were used, which consist of morpholinos modified by covalent binding of an octaguanidin dendrimere allowing to increase AON biodistribution in vivo [32]. Intravenous administration of these AONs in mice led to reduced Progerin amounts, marked amelioration of their progeroid phenotypes and lifespan extension [25]. These results represented an unprecedented and fundamental proof of concept in the field of Progeria therapeutics and suggest that splicing modulation using AONs might be tested in a future clinical trial. In order to expand the cohort of progeroid patients eligible to future treatment by AON, we tested the efficacy of this approach to block the aberrant and pathological splicing in cells from other patients with HGPS-like or mandibuloacral dysplasia with type B lipodystrophy (MAD-B) [17].

## 2. Materials and Methods

### 2.1. Patients and Samples

Samples were collected from seven patients affected with typical HGPS, HGPS-like or MAD-B syndromes, showing variable clinical phenotypes and disease severity but having in common the accumulation of aberrant Prelamin A isoforms. Patients were from France (HGPS-L5), Greece (HGPS-L3), UK (HGPS-L2 and HGPS-L4), USA (HGPS-L1) and Togo (MAD-B). Informed consents were obtained from the patients or the parents of minor patients included in this work, allowing studies on their cells as part of a diagnostic and research program, complying with the ethical guidelines of the institutions involved. Parents also gave written consent for picture publication. The dermal fibroblast cell line from patient HGPS-L 1 was provided by the Progeria Research Foundation Cell and Tissue Bank under the cell line name PSADFN386 (www.progeriaresearch.org); the other human dermal fibroblast cell lines were issued from a skin biopsy, prepared and stored by the labeled Biological Resource Center (CRB TAC), Department of Medical Genetics, Timone Hospital of Marseille (A. Robaglia-Schlupp and K. Bertaux) according to the French regulation. The fibroblast cell lines used belong to a biological sample collection declared to the French ministry of Health (declaration number DC-2008-429) whose use for research purposes was authorized by the French ministry of Health (authorization number AC-2011-1312). The fibroblasts cell lines were cultured in Dulbecco’s modified Eagle’s medium (Life Technologies, Waltham, MA, USA) supplemented with 15% fetal bovine serum (Life Technologies), 2 mm l-glutamine (Life Technologies) and 100 U/mL of penicillin, streptomycin and amphotericin B mix (Life Technologies) at 37 °C in a humidified atmosphere containing 5% CO_2_.

### 2.2. Delivery of Morpholino Oligonucleotides

For morpholino delivery, we used the Endoporter system and followed the manufacturer’s instructions (Gene Tools, LLC, Philomath, OR, USA). Briefly, cells were plated at 50% subconfluence in DMEM (Life Technologies) containing 10% fetal bovine serum (Life Technologies) and 2 mm l-glutamine (Life Technologies) at 37 °C in a humidified atmosphere of 5% CO_2_. Each morpholino oligonucleotide was added at a final concentration of 20 µM to cell cultures in six-well plates. Endoporter reagent was added at a final concentration of 6 µM. Cells were retransfected 48 h later. The effect of morpholino oligonucleotides was assayed 96 h after the first transfection. All transfection experiments were repeated three times.

### 2.3. Morpholino Sequences

Combinations of morpholino oligonucleotides sequences were personalized for each patient’s *LMNA* genotype as described in Table 1. Indeed, as previously mentioned, Lamins A/C are generated by alternative splicing of the *LMNA* gene. The use of a donor splice site within exon 10, generates the Prelamin A-encoding transcripts. A common SNP (dbSNP rs4641: C > T) is located at this alternative splice site [33,34,35], at position c.1698 of the Prelamin A-encoding transcript (NM_170707.3) and was contained in the Ex10 morpholino target sequence (indicated in bold in Table 1). The rs4641 allele segregating in cis on the mutated chromosomes of HGPS-like patients was determined from each patient’s cell line by Sanger sequencing of aberrant Prelamin A transcripts, amplified by RT-PCRs spanning exons 10 and 11, in order to be able to design for each patient the most efficient Ex10 morpholino AON targeting the mutated pre-mRNAs. For the MAD-B patient, for whom rs4641 was homozygous “C” at the genomic level, only the morpholino directed against exon 10 and targeting all the Prelamin A transcripts was used (Table 1). For HGPS-L4 a specific morpholino directed against Prelamin A Δ35 was designed and used in combination with Morpholino-Ex10.

### 2.4. RNA Isolation, Transcriptional Analysis and Real-Time Quantitative PCR

Total RNAs were isolated from cultured cells using RNeasy plus extraction kit (Qiagen, Valencia, CA, USA) and the samples were quantified and evaluated for purity (260 nm/280 nm ratio) with a NanoDrop ND-1000 spectrophotometer. Five hundred nanograms of RNA was reverse transcribed using the High Capacity cDNA Reverse Transcription Kit (Applied Biosystems, Waltham, MA, USA), using random hexamers. PCR amplifications were performed with 2 µL of RT-product with specific primers using the following program: a denaturation step at 95 °C for 3 min, followed by 30 cycles including 95 °C for 30 s, 60 °C for 30 s and finally 72 °C for 45 s and a final elongation step of 3 min. PCR products were separated by electrophoresis in a 2% agarose gel containing ethidium bromide. Primers used for Lamin transcripts amplification were located in exons 9 and 12: forward (5′-GTGGAAGGCACAGAACACCT-3′) and reverse (5′-GTGAGGAGGACGCAGGAA-3′). To analyze the effect of antisense oligonucleotides on β-actin and GAPDH the following primers were used: β-actin ex4-ex5F: 5′-CGCGAGCACAGAGCCTCG-3′; β-actin ex4-ex5R: 5′-TCTTCTCGCGGTTGGCCTTG-3′; β-actin ex3-ex4F: 5′-ACCCAGATCATGTTTGAGAC-3′; β-actin ex3-ex4R: 5′-GGCAGTGATCTCCTTCTGC-3′; β-actin ex1-ex3F: 5′-CCAGCACAATGAAGATCAAGATC-3′; β-actin ex1-ex3R: 5′-TTCAACTGGTCTCAAGTCAGTG-3′; GAPDH ex7-ex9F: 5′-TCCTCCTGTTCGACAGTCAG-3′; GAPDH ex7-ex9R: 5′-AGTTGGTGGTGCAGGAGGC-3′; GAPDH ex1-ex7F: 5′-CCTGGCCAAGGTCATCCATG-3′; GAPDH ex1-ex7R: 5′-GTACTTTATTGATGGTACATGAC-3′.

Quantitative RT-PCR (qRT-PCR) was carried out with the TaqMan Gene Expression Master Mix on a LightCycler 480 (Roche, Berlin, Germany) using pre-designed primers for GAPDH (Hs00266705_g1), Progerin (F: ACTGCAGCAGCTCGGGG. R: TCTGGGGGCTCTGGGC and probe: CGCTGAGTACAACCT), Lamin A (F: TCTTCTGCCTCCAGTGTCACG. R: AGTTCTGGGGGCTCTGGGT and probe: ACTCGCAGCTACCG), Lamin C (F: CAACTCCACTGGGGAAGAAGTG. R: CGGCGGCTACCACTCAC and probe: ATGCGCAAGCTGGTG), Prelamin A Δ90 (F: CGAGGATGAGGATGGAGATGA. R: CAGGTCCCAGATTACATGATGCT, overlapping exons 10 and 12 and probe: CACCACAGCCCCCAGA) (Applied Biosystems) using the program: UNG incubation at 50 °C for 2 min, initial denaturation at 95 °C for 10 min, 45 cycles of amplification: denaturation at 95 °C for 15 s and annealing at 60 °C for 1 min. All PCR reactions were performed in triplicate. Threshold cycle (Ct) values were used to calculate relative mRNA expression by the 2-ΔΔCT relative quantification method using normalization to GAPDH expression and EP condition as the reference value for each patient.

### 2.5. Western-Blot

Total fibroblast proteins were extracted in 200 μL of NP40 Cell Lysis buffer (Invitrogen, Carlsbad, CA, USA) containing Protease and Phosphatase Inhibitor Cocktail (Thermo Fisher Scientific Inc. Waltham, MA, USA). Cells were sonicated twice (30 s each), incubated at 4 °C for 30 min and then centrifuged at 10,000 g for 10 min. Protein concentration was evaluated with the bicinchoninic acid technique (Pierce BCA Protein Assay Kit), absorbance at 562 nm is measured using nanodrop 1000 (Thermo Fisher Scientific Inc. Waltham, MA, USA) Equal amounts of proteins (40 µg) were loaded onto 10% Tis-Glycine gel (CriterionTM XT precast gel) using XT Tricine running Buffer (Biorad, Hercules, CA, USA). After electrophoresis, gels were electro transferred onto nitrocellulose membranes or Immobilon-FL polyvinylidene fluoride membranes (Millipore), blocked in Odyssey Blocking Buffer diluted 1:1 in PBS for 1 h at room temperature, and incubated overnight at 4 °C or 2 h at room temperature with various primary antibodies diluted in blocking buffer added with 0.1% Tween 20: 1:1000 rabbit polyclonal anti-Lamin A/C (sc-20681, Santa Cruz Biotechnology, Dallas, TX, USA), 1:200 goat polyclonal anti-Lamin A/C (sc-6215, Santa Cruz Biotechnology), 1:1000 mouse monoclonal anti-Progerin (ab66587, Abcam, Cambridge, UK), 1:1000 mouse monoclonal anti-actin (MAB1501, Merck Millipore, Darmstadt, Germany) and 1:40,000 monoclonal anti-glyceraldehyde-3-phosphate dehydrogenase (GAPDH) (MAB374, Merck Millipore). Blots were washed with TBS-T buffer [20 mM tris (pH 7.4), 150 mm NaCl, and 0.05% Tween 20] and incubated with 1:10,000 IR-Dye 800-conjugated secondary donkey anti-goat or IR-Dye 700-conjugated secondary anti-mouse antibodies (LI-COR Biosciences) in blocking buffer added with 0.1% Tween 20 (LI-COR Biosciences, Lincoln, NE, USA). For IR-Dye 800 and IR-Dye 700 detection, an Odyssey Infrared Imaging System (LI-COR Biosciences) was used. GAPDH, α-tubulin or β-actin were used as a total cellular protein loading control.

### 2.6. Senescence Assay

Senescence was measured with a Beta-Glo Assay kit (Promega), according to the manufacturer’s instructions. Luminescence intensity was determined as relative light units (RLUs) using a GloMax-Multi Detection System: Luminometer (Promega, Madison, WI, USA).

### 2.7. Quantitation of Abnormal Nuclear Morphology

Fibroblasts from HGPS and HGPS-like patients were cultured with DMEM medium containing 20 µM AON, or 6 µM of vehicle (Endoporter) for 96 h. Cells stained for Lamin A/C or DAPI were examined by fluorescence microscopy with an Axioplan 2 imaging microscope (Carl Zeiss, Oberkochen, Germany). For quantitation of abnormal nuclear morphology, the percentage of normal nuclei (nuclei with a smooth oval shape) and abnormal nuclei (nuclei with blebs, irregular shape, or multiple folds) was calculated using the Nuclear Irregularity Index (NII) plugin of the Image J Software (Version 1.6.0, NIH, USA). The analysis uses measures of nuclear area and of four parameters of irregularity, named Aspect, Area/Box, Radius Ratio and Roundness. These four parameters are used to generate a Nuclear Irregularity Index (NII) which, added to area measurement, classify the nuclei in normal or abnormal morphology. At least 100 fibroblast nuclei were randomly selected for each cell line.

### 2.8. Statistical Analyses

Statistical analyses were performed with GraphPad Prism 6 (GraphPad Software, Inc. San Diego, CA, USA). Differences between groups were assayed using a two-tailed Student’s t-test. In all cases the experimental data were assumed to fulfill t-test requirements (normal distribution and similar variance); in those cases, where the assumption of the t-test was not valid, a non-parametric statistical method was used (Mann–Whitney test). A *p* value less than 0.05 was considered as significant. Error bars indicate the standard error of the mean.

## 3. Results

### 3.1. Patients’ Molecular and Clinical Features

**Figure 1:**
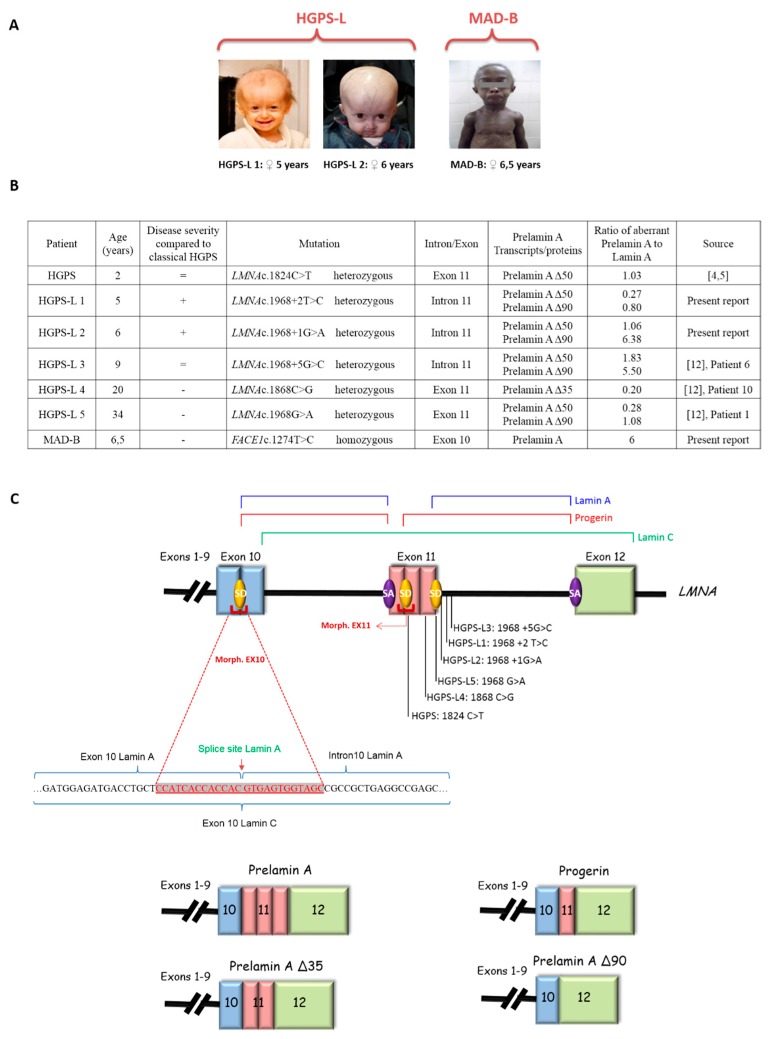
Clinical and molecular characterization of the patients. (**A**) Facial features of Hutchinson-Gilford Progeria Syndrome-like (HGPS-like) and Mandibuloacral Dysplasia type B (MAD-B) patients reveal a spectrum of progeroid clinical features including alopecia or sparse hair, thin and transparent skin with loss of subcutaneous fat and prominence of subcutaneous vessels, broad forehead with frontal bossing. (**B**) Molecular characterization of *LMNA* and *FACE1(ZMPSTE24)* gene mutations in HGPS-like and MAD-B patients eliciting aberrant Prelamin A splicing or wild type Prelamin A accumulation. Variable disease severities compared to classical HGPS are indicated with “+”: more, “−“: less, or “=”: equal severity. The ratios of aberrant Prelamin A to Lamin A isoforms are shown, issued from Western Blot data, except for Prelamin A against which no antibodies are available and so were determined based on the transcript levels. (**C**) Schematic representation of the aberrant Prelamin A isoforms and the morpholino-based strategy for *LMNA* splicing modulation. SD: Splice Donor site, SA: Splice Acceptor site.

Patients included in this study showed variable disease severity compared to classical HGPS but presented with similar phenotypes, including poor growth, hair loss, prominent forehead, prominent superficial veins, thin skin, loss of subcutaneous fat and lipodystrophy (Figure 1A). HGPS-like patients were previously reported to present distinct aberrant splicing patterns of Prelamin A pre-mRNAs due to mutations located around exon 11 donor splice site (Figure 1B,C). Patient HGPS-L1 carrying the *LMNA* heterozygous c.1968 + 2T > C mutation was referred to our center at age five years old. She was diagnosed with the disease when she was 10 months old, presenting with a typical HGPS clinical phenotype, including frontal bossing, prominent veins on her scalp and forehead, sparse hair, micrognathism with delayed dentition, growth retardation (since birth, her length varied from the 2nd to the 10th centiles for age; the weight was stably <3rd centile for age), subcutaneous lipoatrophy, dry skin with pigmentary changes on the neck and trunk, acroosteolyses with onychodystrophy of hands and feet; laboratory findings have evidenced recurrent thrombocytosis (480–535 k/µL; normal values: 140–450 k/µL), elevated transaminases, glucose, calcium and phosphorus, as already observed in classical HGPS patients [36]. HGPS-L2 patient, carrying the heterozygous *LMNA* c.1968 + 1G > A mutation, showed a very similar progeroid laminopathy, though evolving more severely. She was diagnosed at nine months of age and already showed contractions of her ankles, knees, and wrist. She subsequently developed arthritis on several articulations. Her feeding was poor and she had frequent constipation episodes. She had bilateral hip dislocation and at the age of three years she suffered from a femur fracture. At age six, she suffered from tachycardia together with sudden right arm paresis; cerebral CT scan/MRI showed multiple micro-infarcts, including recent and old ones, while echocardiography showed left ventricular thickening. After partial recovery from stroke, she suffered from a chest infection together with painful nails’ infections. Patients HGPS-L3 (*LMNA* heterozygous c.1968 + 5G > C), HGPS-L4 (*LMNA* heterozygous c.1868C > G) and HGPS-L5 (*LMNA* heterozygous c.1968G > A) were previously reported by Barthelemy et al. [12]. The MAD-B patient was first referred to us at age 6.5 years. She presented with growth retardation, exophtalmia, low-set ears, retro-micrognathism (mandibular hypoplasia), sparse hair and thin, dry skin with hypopigmented lesions, especially on the trunk. Subcutaneous lipoatrophy gave her a muscular pseudo-hypertrophic appearance. Molecular genetic diagnosis allowed the identification of a new homozygous mutation in the *ZMPSTE24/FACE1* gene’s exon 10: c.1274T > C, p.(Leu425Pro), confirming the B-type Mandibuloacral dysplasia phenotype in the patient [19].

### 3.2. Efficient Reduction of Aberrant Pre-mRNA Splicing in HGPS-Like and MAB-B Patient Cells Using Antisense Morpholinos

To evaluate the effects of antisense morpholinos in reducing aberrant *LMNA* splicing, we treated the primary fibroblasts cell lines of five patients with HGP-like syndromes, one patient with HGPS and one patient with MAD-B, following the same protocol described in [25] and adapted depending on the splice donor site and the genotype of the patient at dbSNP rs4641.

**Figure 2:**
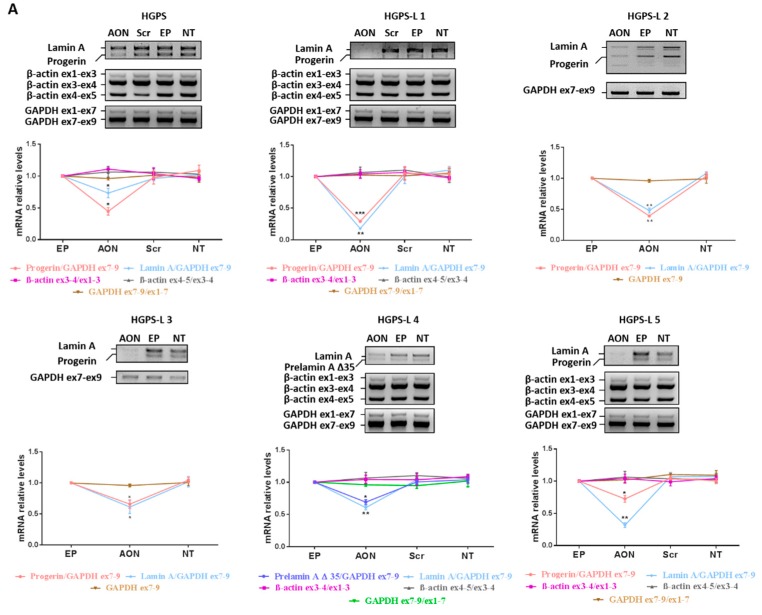

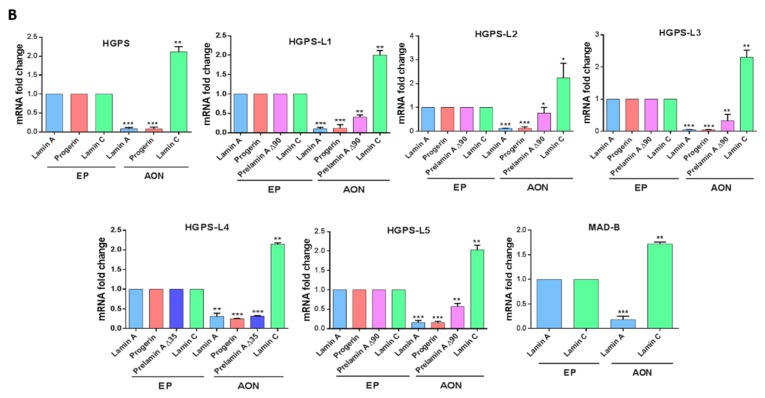
Morpholino treatment downregulates aberrant Prelamin A transcripts production without causing aberrant splicing in off-target genes. (**A**) Upper panels: Semi-quantitative RT-PCR analyses showing Lamin A, Progerin (Prelamin A Δ50), Prelamin A Δ35, β-actin and GAPDH mRNA levels in Hutchinson-Gilford Progeria Syndrome (HGPS) and HGPS-like fibroblasts treated with morpholino antisense oligonucleotides (AON, 20 µM each) relative to Endo-porter (EP, which is the AON vehicle used as Control), scrambled oligonucleotides (Scr) treated cells and untreated cells (NT). Fragments covering all exons of β-actin (3 fragments, 5 exons) and GAPDH (2 fragments, 9 exons) cDNAs were amplified for each couple of AON used. Given that in patients HGPS-L2 and -L3, the same AON couple as in patient HGPS-L1 was used, the splicing study was only partially replicated. Lower panels: Relative levels of Lamin A, Progerin, Prelamin A Δ35 to GAPDH ex7–ex9 and the indicated fragments of β-actin and GAPDH transcripts ratios using Image J software and compared to EP treated cells. (mean ± SEM, *n* = 4, Student’s t-test, * *p* < 0.05, ** *p* < 0.01, *** *p* < 0.001, AON-treated vs. EP-control). (**B**) Quantitative RT-PCR analyses of Lamin A, Progerin (Prelamin A Δ50), Prelamin A Δ35, Prelamin A Δ90 (when detected at baseline, in EP assays), Lamin C and GAPDH mRNA levels in HGPS, HGPS-like and Mandibuloacral Dysplasia type B (MAD-B) fibroblasts treated with AON, 20 µM each relative to Endoporter (EP: vehicle used as Control). The fold change of each transcript was determined by normalizing its value to that of GAPDH for each condition. (mean ± SEM, *n* = 4, Student’s t-test, * *p* < 0.05, ** *p* < 0.01, *** *p* < 0.001, experimental vs. control).

As shown in Figure 2A, the effect of antisense morpholinos was first assessed at the transcriptional level by semi-quantitative RT-PCR using primers flanking the region between exons 9 and 12 of the LMNA gene (cf. Section 2.4) and generating amplicons that were, respectively, 510 bp (Lamin A), 405 bp (Prelamin A Δ35) and 360 bp (Prelamin A Δ50). The results show that treatment of cells either with scrambled AON or with Endoporter had no effect on the levels of aberrant Prelamin A transcripts, which were similar to non-treated (NT) samples and did not result in generation of any aberrant splice products for β-actin and GAPDH suggesting that the observed response was specific to the personalized morpholino AON. For the cell lines of patients HGPS-L2 and -L3, the exploration was not entirely replicated since the AON used were exactly the same as for patient HGPS-L1, in whom no aberrant splicing was evidenced (cf. Table 1). When compared to control cells treated with Endoporter (EP), the analysis of aberrant transcripts’ amounts upon morpholino treatment revealed clear reductions in Lamin A and Prelamin A Δ50 mRNA in HGPS, HGPS-L1, HGPS-L2, HGPS-L3 and HGPS-L5 patients’ cells and Prelamin A Δ35 mRNA in HGPS-L4 patient cells. We obtained similar results when we analyzed the amounts of these aberrant isoforms, including Prelamin A Δ90, in morpholino-treated cells using quantitative RT-PCR (Figure 2B). Indeed, deleted Prelamin A transcripts were greatly reduced, and the one corresponding to Lamin C was increased, as expected, due to the directed splicing shift of LMNA pre-mRNAs, favoring Lamin C production at the expense of Lamin A isoforms. The treatment also significantly decreased the production of Lamin A transcripts in MAD-B fibroblasts.

### 3.3. Antisense Morpholinos Reduce Aberrant Prelamin A Levels in HGPS-Like and MAB-B Patient Cells

**Figure 3:**
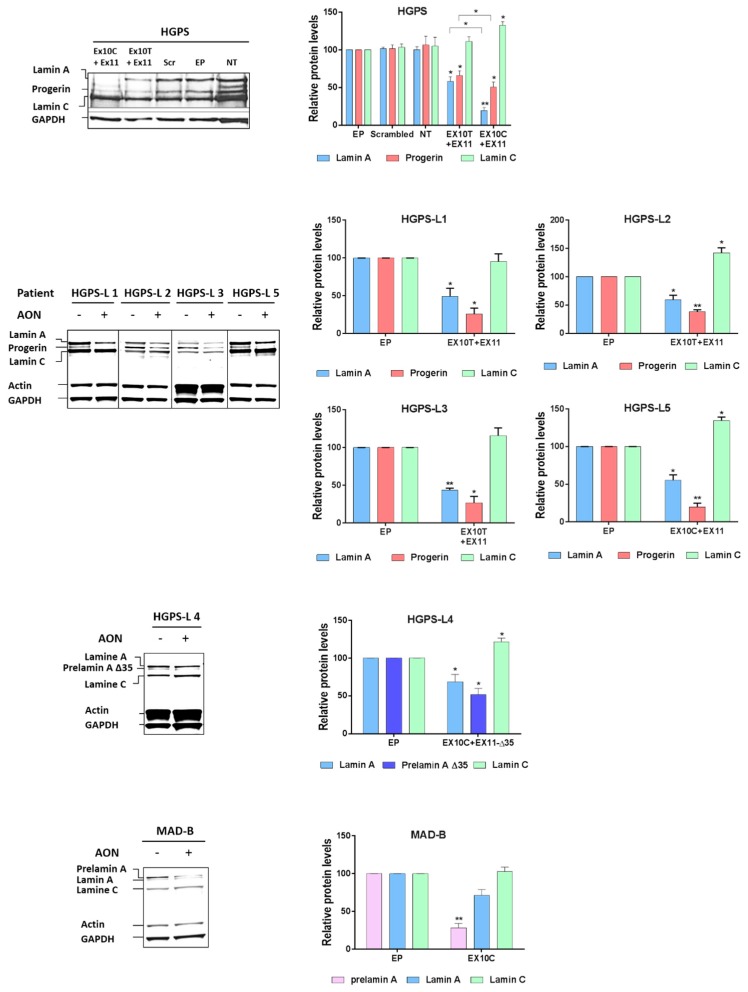
Antisense morpholinos reduce aberrant Prelamin A protein levels in Hutchinson-Gilford Progeria Syndrome-like (HGPS-like) and Mandibuloacral Dysplasia type B (MAB-B) patient cells. Left panels: Western blotting evaluation of Lamin A/C, Progerin, Prelamin A, Prelamin A ∆35, actin and GAPDH in whole lysates from HGPS, HGPS-like and MAD-B fibroblasts treated with Endoporter (−) (control), antisense morpholinos: AON (+). Right panels: Lamin A/C, Progerin, Prelamin A, Prelamin A ∆35 expression levels were normalized to GAPDH values using ImageJ software. (mean ± SEM, *n* = 3, Student’s t-test, * *p* < 0.05, ** *p* < 0.01. AON-treated vs. control, EP-treated cells).

The significant reduction of aberrant Prelamin A mRNA levels prompted us to evaluate the corresponding protein levels upon morpholino AON treatment. As shown in Figure 3, we observed that treatment of patients’ fibroblasts with morpholinos led to a marked decrease of all aberrant Prelamin A protein levels relative to Endoporter treated cells (control) in all patients, including the MAD-B patient.

Moreover, given the heterozygous status of the LMNA rs4641 allele in the HGPS patient, carrying the rs4641 “C” genotype in cis of the c.1824C > T mutation, we showed that the use of morpholino-Ex10 “C allele” was about 25% more effective in reducing Progerin levels than the Ex10-morpholino “T allele”. Similarly, we have shown that treatments with scrambled AON as well as with the Endoporter had no effect on Prelamin A levels, indicating the specific action of the personalized morpholino AON in reducing Prelamin A isoforms’ expression.

### 3.4. Antisense Morpholinos Reduce Senescence in HGPS-Like and MAB-B Patient Cells and Improve Nuclear Shape Abnormalities

**Figure 4:**
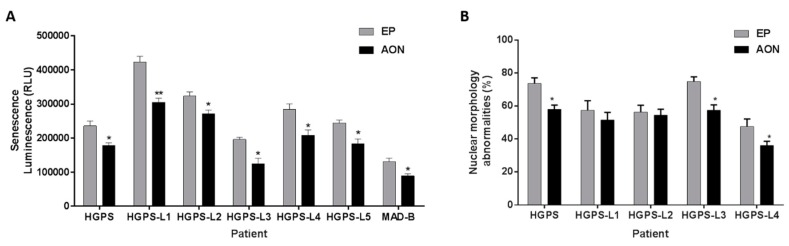
Antisense morpholinos reduce senescence in Hutchinson-Gilford Progeria Syndrome (HGPS), HGPS-like and Mandibuloacral Dysplasia type B (MAD-B) cell lines and improves nuclear shape abnormalities. (**A**) Senescence levels in HGPS, HGPS-like and MAD-B fibroblasts treated with antisense morpholinos (AON) for 10 days, relative to control, Endoporter-treated cells (EP). Senescence is determined as relative light units (RLU); bars show mean ± SEM, *n* = 3, Student’s t-test, * *p* < 0.05, ** *p* < 0.01). (**B**) The percentage of abnormal nuclei (nuclei with blebs, irregular shape, or multiple folds) of HGPS and HGPS-like fibroblasts treated with 20 µM of each antisense morpholino (AON) for 96 h, relative to control, i.e., Endoporter-treated cells (EP), was calculated using the Nuclear Irregularity Index (NII) plugin of the Image J Software. The mean values of three different experiments are shown. * *p* < 0.05.

Finally, since cellular senescence is considered as a major hallmark of HGPS, we assessed senescence levels, measured by β-galactosidase quantification, upon morpholino AON treatment in HGPS, HGPS-like and MAD-B fibroblasts. As shown in Figure 4A, in all the tested cell lines, senescence-associated β-galactosidase activity levels were decreased by a 10-day-long morpholino treatment, when compared to the same cell lines treated the same number of days with Endoporter. In addition, Using Lamin A/C and DAPI staining, the characteristic nuclear defects have been explored to determine the effectiveness of AON treatments towards reversing HGPS and HGPS-like nuclear abnormalities. As shown in Figure 4B, among the cells tested, HGPS, HGPS-L3 and HGPS-L4 exhibited a significant reduction in nuclear blebbing compared with the passage-matched and Endoporter-treated cells.

## 4. Discussion

We previously showed that the reduction of Progerin levels can be achieved via an antisense therapeutic approach, with beneficial effects both in vitro on human HGPS fibroblasts and *Lmna^G609G/G609G^* fibroblasts and in vivo on the *Lmna^G609G/G609G^* mouse model [25], establishing the first in vivo proof of principle of efficacy for a future trial based on antisense approaches in HGPS. Recently, similar results were obtained by another group on the same *Lmna^G609G/G609G^* mouse model, confirming the efficacy of this approach [37]. However, *LMNA* mutations other than the classical p.G608G have been shown to cause the production of Progerin and/or other truncated Prelamin A isoforms in patients affected with HGPS-like syndromes [12,13,14,15,16]. These patients are theoretical candidates to enter into the same kind of clinical trial, since they share the same pathophysiological mechanism based on abnormal pre-mRNA splicing, leading to the accumulation of Progerin and/or other truncated Prelamin A isoforms. Towards this end, obtaining further in vitro preclinical evidence of benefit and efficacy of such an approach by adapting the AON treatment to the different genotypes was indicated. In this study, we assessed this treatment’s efficacy in reducing the production of all Lamin A isoforms, notably including aberrantly accumulated Prelamin A isoforms, either truncated or wild type, in HGPS-like and MAD-B patients’ cells. The treatment’s efficacy was analyzed at the transcriptional and protein levels in all HGPS-like patients. We show a significant decrease of each aberrant transcript’s production (Prelamin A Δ35, Δ50 and Δ90) and also of the corresponding abnormal proteins, excepted for Prelamin A ∆90 against which there is no specific antibody and whose size is equal to that of Lamin C, thus making it unidentifiable using an anti-Lamin A/C antibody. Nonetheless, this issue is of little concern since previous studies using mass spectrometry in cell lines of patients producing high levels of the Prelamin A ∆90 transcript failed to evidence the production of a peptide corresponding to this isoform, suggesting that its transcript is not or very faintly translated [12]. A MAD-B patient, carrying the homozygous missense mutation p.Leu425Pro in the FACE1/ZMPSTE24 gene, was also included in this study, in order to expand the cohort of patients eligible to treatment by the antisense approach.

In all patients, AON treatment elicited Lamin C transcripts’ upregulation. This increase, observed by quantitative RT-PCR, was expected. Indeed, these transcripts are produced from pre-mRNAs encoded by the *LMNA* gene when the splice site within exon 10 leading to the production of Prelamin A isoforms is not used. In most cases, although depending on organism and tissues, the production of Lamin A vs. Lamin C isoforms is physiologically relatively balanced (with the exception of neural cells [38,39]); in our work, the steric blockade of the exon 10 splice site forces a shift in the splicing balance towards Lamin C-encoding transcripts. Based on previous preclinical data, it is expected that Lamin C can, at least partially, compensate the absence of Lamin A since mice expressing Lamin C only show no phenotypic abnormality and even an extended lifespan [25,40,41]. Anyway, considering the severe phenotypes of HGPS, HGPS-like and MAD-B patients, the benefit/risk balance is undoubtedly in favor of reducing toxic isoforms of Prelamin A. Further supporting the view of a favorable effect induced by Prelamin A reduction, we provided evidence that prolonged treatment with morpholino AONs also had beneficial functional consequences, shown by a reduced number of cells expressing the senescence marker β-galactosidase and reducing the typical nuclear blebbing in some of the tested cell lines after a short-term treatment.

Importantly, several preclinical results or patient-extrapolated data support the idea that even relatively small reductions in the amounts of accumulated Prelamin A may have important clinical/phenotypic beneficial consequences, making this approach very promising in terms of disease prevention/recovery [42].

The spectacular absence of pathological phenotypes in *Zmpste24^−/−^* *Lmna^+/−^* mice, protecting them from all of the pathological consequences of Zmpste24 deficiency and the disease phenotypes of Progeria [43], suggests that reducing by half the quantities of accumulated Prelamin A may be the gold standard for treatment efficacy. Nonetheless, the prevention of progeroid phenotypes upon treatment with antisense morpholino oligonucleotides was demonstrated in vivo even if the reduction of the amounts of Progerin (protein) accumulated in mice tissues was relatively low [25]. Similar beneficial results with very limited reduction of Progerin amounts in vivo was recently confirmed [37]. Furthermore, other studies suggest that at least certain pathological phenotypes (e.g., of bone) may be even reverted upon lowering the expression of Progerin [42].

AON chemistries are also continually evolving with increasing bioavailabilities and biodistribution properties; e.g., tricyclo-DNA oligomers enable dystrophin rescue and functional improvement in all tissues affected by the lack of dystrophin in mouse DMD models [44]. Finally, a splicing modulation strategy (exon skipping) using morpholino AONs was successfully already used in pediatric populations, in the context of Duchenne muscular dystrophy [45,46], making the use of AON with similar chemistry more secure.

Overall, the choice of AON chemistry and route of administration will be particularly important for future therapeutic approaches involving splicing-modulation by antisense oligonucleotides in diseases that are amenable to this approach like HGPS and related syndromes. The results we report establish a preclinical proof of principle for the use of antisense morpholino oligonucleotides in HGPS-like and MAD-B syndromes, further extending, besides classical HGPS, the eligibility of patients for the inclusion in a future trial based on this therapeutic approach.

## Figures and Tables

**Table 1:** Morpholino oligonucleotides’ sequences used in this study depending on each patient’s *LMNA* genotype at exons 10 and 11 target sites.

Patient	rs4641 Allele (Verified on the Aberrant Prelamin A cDNA)	Morpholino Name	Morpholino Sequence
HGPS	C	Ex10-allele C	5′-GCTACCACTCACGTGGTGGTGATGG-3′
		Ex11-HGPSmut	5′-GGGTCCACCCACCTGGGCTCCTGAG-3′
HGPS-L1	T	Ex10-allele T Ex11	5′-GCTACCACTCACATGGTGGTGATGG-3′ 5′-GGGTCCGCCCACCTGGGCTCCTGAG-3′
HGPS-L2	T		
HGPS-L3	T		
HGPS-L4	C	Ex10-allele C	5′-GCTACCACTCACGTGGTGGTGATGG-3′
		Ex11-Δ35	5′-ACTGACCGTGACACTGGAGGCAGAA-3′
HGPS-L5	C	Ex10-allele C	5′-GCTACCACTCACGTGGTGGTGATGG-3′
		Ex11	5′-GGGTCCGCCCACCTGGGCTCCTGAG-3′
MAD-B	Homozygous C	Ex10-allele C	5′-GCTACCACTCACGTGGTGGTGATGG-3′
Negative controls	-	Ex10-scrambled	5′-ATCGGCTTGTCGCGTGAGCGATCGA-3′
		Ex11-scrambled	5′-ACCAGTGGCGTCGCCTCGCAGGTCC-3′
